# Predicting cascading extinctions and efficient restoration strategies in plant–pollinator networks via generalized positive feedback loops

**DOI:** 10.1038/s41598-023-27525-3

**Published:** 2023-01-17

**Authors:** Fatemeh Sadat Fatemi Nasrollahi, Colin Campbell, Réka Albert

**Affiliations:** 1 Department of Physics, Pennsylvania State University, University Park, 16803 USA; 2 Department of Biochemistry, Chemistry, and Physics, University of Mount Union, Alliance, 44601 USA; 3 Department of Biology, Pennsylvania State University, University Park, 16803 USA

**Keywords:** Complex networks, Computational science, Ecological networks, Computational biophysics, Functional clustering

## Abstract

The extinction of a species in a plant–pollinator mutualistic community can cause cascading effects and lead to major biodiversity loss. The ecologically important task of predicting the severity of the cascading effects is made challenging by the complex network of interactions among the species. In this work, we analyze an ensemble of models of communities of plant and pollinator species. These models describe the mutualistic inter-species interactions by Boolean threshold functions. We show that identifying generalized positive feedback loops can help pinpoint the species whose extinction leads to catastrophic and substantial damage to the whole community. We compare these results with the damage percentage caused by the loss of species identified as important by previously studied structural measures and show that positive feedback loops and the information gained from them can identify certain crucial species that the other measures fail to find. We also suggest mitigation measures for two specific purposes: (1) prevent the damage to the community by protecting a subset of the species, and (2) restore the community after the damage by restoring a subset of species. Our analyses indicate that the generalized positive feedback loops predict the most efficient strategies to achieve these purposes. The correct identification of species in each category has important implications for conservation efforts and developing community management strategies.

## Introduction

Communities of species are supported by numerous interactions among the species. Elucidating the relationship between inter-species interactions and the resultant repertoire of community compositions has been of general interest^1–4^. Representation of communities as networks and network-based dynamical modeling have been two of the most powerful methods in the field of computational ecology. The interactions among species include competition for common resources, predator-prey interactions, and mutualistic interactions (e.g., pollination and seed dispersal). Network-based studies of ecological systems range from the statistical analysis of food webs^5–8^, to network models of communities in which species have mutualistic and competing interactions^9^, and the use of multi-layer networks to incorporate several types of interactions simultaneously^10,11^.

Here we study plant–pollinator mutualistic interactions that lead to sustainable communities of species. While there are considerable efforts to maintain the biodiversity in such systems, ecological systems and specifically pollinator species still face degradation across the world. The loss of pollinator species has a dramatic negative effect on crops^12–15^ as the majority of food crops require pollination to survive^16^. These communities are subject to various types of perturbation from natural causes to human interference; hence the study of their reliability and stability is of crucial importance for agricultural management and ecological preservation efforts^8,17–19^.

A cascading effect and major biodiversity loss can result from the extinction of a species in a plant–pollinator mutualistic community. The complex set of interactions among the species challenges the estimation of the severity of the cascading effects and the development of restoration strategies. Bipartite networks are widely used to represent plant–pollinator communities; in such networks the two types of nodes denote the plant and pollinator species, and each edge denotes the interaction between a plant and a pollinator^20^. This network representation is also implemented to study species extinction and develop possible restoration strategies based on network measures^21^. Studies have also used previously established network measures and newly developed algorithms to identify the keystone species whose extinction causes catastrophic damage to the plant–pollinator communities (e.g., see^22^). In this study we aim to analyze species extinction and community restoration in network ensembles generated by the well-established Campbell et al. model^23^ of plant–pollinator community assembly.

The Campbell et al. model uses two key assumptions to dynamically model plant–pollinator community assembly: First, there is an influx of species from a regional species pool, simulated as recurring invasion attempts, and second, species persist due to receiving enough benefit from their interactions with each other. The regional source pool of species is represented by a bipartite network encoding the mutualistic interactions; while this pool is not necessarily representative of a stable community, a subset of species might be able to form and sustain a community. These possible stable communities are identified as the attractors (long-term behaviors) of the dynamical model.

The response of such communities to perturbations has been well-studied; for instance, the effect of global and local species loss^24,25^, invasion of new species^26^, and translocation of species as a means for community restoration^27^ on the stable communities have been characterized. In this work, we contribute to this line of inquiry by identifying the species whose extinction causes considerable cascading damage to the communities. We show that the information gained from generalized positive feedback loops can best estimate the magnitude of this damage. We also study community restoration and use these positive feedback loops to identify the species whose re-introduction is most beneficial to the community.

In our previous work, we characterized the relationships between generalized positive feedback loops and showed that these relationships determine the number of alternative stable communities^28^. In this study we use these relationships to identify the key species in each of the cases of species extinction and community restoration. Specifically we seek to answer three main questions: (1) Which are the keystone species whose extinction leads to community collapse? (2) How and to what extent can we prevent cascading species extinction once we identify these keystone species? (3) If cascading extinction occurs, how and to what extent can we restore the community? We show that the knowledge gained from generalized positive feedback loops provides substantial insight to answer these questions. We also demonstrate that the study of positive feedback loops has advantages over previously studied methods in community management research.

### Background on the Campbell et al. model

This discrete-time Boolean network model describes plant–pollinator community formation^23^. In this model, plant and pollinator species are represented by two different types of nodes. The habitual visitation of a plant by a pollinator is represented by two directed edges of opposite directions between the two nodes representing the interacting species. The nature of the interaction depends on the degree of the match between the length of the pollinator’s proboscis ($l_{po}$) and the plant’s corolla depth ($l_{pl}$). There are two scenarios of possible mismatch: (i) If $l_{pl}>1.1l_{po}$ the pollinator is in physical contact with the plant (contributing to its pollination), but is not able to feed from the plant nectar. The time and effort spent on visiting the plant is wasted; hence this type of interaction is represented as a positive edge toward the plant and a negative edge toward the pollinator. (ii) If $l_{pl}<0.9l_{po}$ the pollinator is able to feed from the plant nectar but does not land on the plant and therefore fails to pollinate it. This type of interaction is represented as a negative edge toward the plant and a positive edge toward the pollinator. Otherwise, there is a high degree of matching and both species benefit from the interaction; both edges are positive.

This model takes advantage of ensembles of prototypical networks to not only reveal the properties of stable plant–pollinator communities, but also study the underlying mechanisms of extinction/restoration processes in such communities. In this model, plant–pollinator interactions are assigned such that the degree distribution follows an exponentially cut-off power law with properties matching empirical data^29^. The characteristic properties of species (plants’ corolla depths and pollinators’ proboscis lengths) are taken from skew normal distributions reported by Stang et al.^30^. Following the assignments of characteristic properties and interactions, only about 7.7% of the interactions in the regional species pool are mutually beneficial, and the rest are beneficial in one direction and detrimental in the other direction. We used 6 ensembles of 50–70 nodes; each ensemble consists of 1000 networks that have the same number of plant and pollinator species, while the degree distribution of the network and the characteristic length assigned to each node is different.

In the Campbell et al. model each species *i* is assumed to be either present (“active”) with the state $\sigma _i(t)=1$, meaning that its population abundance is above a threshold value at time *t*, or absent (“inactive”) with the state $\sigma _i(t)=0$, meaning that its population abundance is below a threshold value (though such a species still exists, and can invade from, the regional species pool). The synchronous update scheme is used in this model, i.e., the states of all nodes are updated at each time step *t*. The state of node *i* at time $t+1$ is determined by the state of its regulators at time *t* via an update function *f*. Specifically,

1 $$\begin{aligned} \sigma _i(t+1)=f_i(\sigma _j(t))=H\Big (\sum _j E(j,i)\sigma _j(t)\Big ), \end{aligned}$$

where $\{\sigma _j\}$ are the states of the regulators of node *i* at time *t*, *E*(*j*, *i*) is the weight of the interaction from node *j* to node *i*, and the step function *H*(*x*) equals 1 if $x>0$ and zero otherwise. According to the update function, each species is required to have a net positive influence from the community to be able to establish or persist at each time step. Each positive interaction benefits the target species more than each negative interaction harms it. The model reflects this by assuming that the weight of a positive interaction is more than the absolute value of the weight of a negative interaction. To keep consistent with the Campbell et al. study, we use a weight of $-1$ for negative interactions and $+4$ for positive interactions (see^23^ for additional details).

### Key concepts of Boolean modeling

The Campbell et al. model is a Boolean model—a model whose variables have binary states. The dynamics of Boolean models is determined by two features: the update function and the implementation of time via an update scheme. The Campbell et al. model is governed by a Boolean threshold function as described in Eq. (1), and originally used synchronous update, where all nodes are updated at multiples of a common time step. There also are non-deterministic update schemes that are more appropriate in certain contexts. In general asynchronous update (also called fully asynchronous update) a single, randomly selected node is updated at each time step. The recently introduced Most Permissive Boolean Networks (MPBNs) include two additional states: increasing and decreasing, stochastic transitions into these states and instantaneous transitions out of them. When projecting the four-state system’s state transitions to the states 0 and 1, all possible update orders (including partial or full synchrony among nodes) are represented^31^.

In a Boolean model every temporal trajectory ends in a set of states in which it is trapped. This set of states is referred to as the attractor of the Boolean model. Attractors in which all nodes have a fixed state (either active or inactive) are called point attractors, steady states, or fixed points. Attractors that contain several states depend on the manner of update of the nodes. Synchronous update leads to a deterministic rotation through states in limit cycles, while stochastic update (e.g., updating a randomly selected node at each time step) leads to so-called complex attractors.

**Figure 1:**
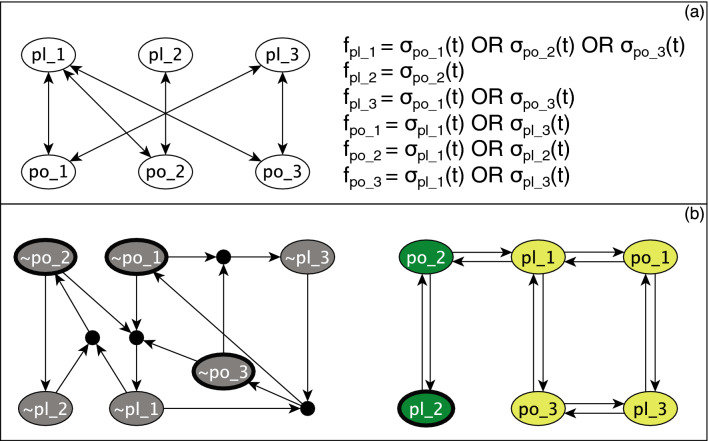
Illustration of Boolean modeling key concepts in the context of a plant–pollinator interaction network. (**a**) The interaction network with the Boolean threshold functions converted to disjunctive prime format. (**b**) The expanded network in which there is a black composite node for each “AND” gate and two virtual nodes representing the possible node states. The active state is denoted by the node name and the inactive state is shown using the name of the node prefixed with $\sim$. The inactive stable motif highlighted with grey corresponds to the full community collapse. One of the multiple driver sets of this stable motif is indicated by solid black outlines. The whole grey stable motif is in the LDOI of this driver set. The stable motif highlighted with dark green is one of the active stable motifs driven by either virtual node pl_2 (outlined) or po_2. The virtual nodes highlighted with light green are stabilized as a result of stabilization of this stable motif, i.e., they are in the LDOI of the dark green stable motif. The LDOI of the dark green stable motif also contains the virtual nodes of the dark green stable motif, as generally true for all stable motifs.

In general, the task of finding the attractors of Boolean models is challenged by the computational complexity caused by the exponential growth of the size of the state space. To resolve this problem, we use stable motif analysis, a novel method that allows for efficient identification of attractors. This method relies on the construction of the expanded network, which encodes the information about the structure of the interaction network and the Boolean functions governing the dynamics^32,33^. In the following we describe the key concepts and methodologies involved in stable motif analysis and illustrate them using an example in Fig. 1.

### Construction of the expanded network

The expanded network expresses the Boolean update function of each node in disjunctive prime form, i.e., the complete sum of prime implicants, also called Blake Canonical form^34^. For each node *i* in the interaction network, there are two virtual nodes in the expanded network, which denote the node’s two possible states: the active state is represented by a virtual node that has the label *i* and the inactive state is represented by a virtual node labeled with $\sim i$. “AND” gates among virtual nodes are represented by composite nodes. Any edge from a virtual node to another virtual node indicates a sufficiency relationship, i.e., the node state described by the source virtual node is sufficient for the node state described by the target virtual node. Any edge from a virtual node to a composite node indicates a necessary relationship, i.e., the node state described by the source virtual node is necessary for the the node state described by the virtual node that the composite node points to. The totality of the inputs of a composite node are together sufficient, as this group of virtual nodes represents a prime implicant of the Boolean update function. In Fig. 1a an interaction network made up of 3 plant and 3 pollinator nodes and the corresponding Boolean functions are illustrated. Panel (b) shows the corresponding expanded network, which contains twelve virtual nodes and four composite nodes (black circles).

### Stable motif analysis

A stable motif is defined in the expanded network such that it is a strongly connected subgraph (a subgraph that has a least one path in both directions for any pair of virtual nodes) that (i) is consistent, meaning that it does not contain a virtual node and its negation, (ii) is composite-closed, i.e., it includes all of the input virtual nodes to the composite nodes inside the component, and (iii) is a minimal subgraph, meaning that no subset of it can satisfy all the above conditions. In Fig. 1b the grey subgraph is a stable motif that represents the inactivity of all its constituent nodes. The dark green subgraph is another stable motif representing the activity of the two nodes involved.

Conditionally stable motifs are weaker counterparts of stable motifs that only stabilize if a specific (set of) condition(s) is satisfied. Specifically, conditionally stable motifs are strongly connected subgraphs of the expanded network such that (i) the subgraph is consistent, i.e., it does not contain a virtual node and its negation, (ii) the subgraph is not composite-closed, i.e., it contains at least one composite node without containing all of its incoming virtual nodes^35^. The external virtual nodes that are the inputs to the composite nodes inside the subgraph make up the conditions of the conditionally stable motif. If during the dynamics of the system, stabilization of a stable motif results in the stabilization of the conditions of a conditionally stable motif, the conditionally stable motif becomes a stable motif. We call such a stable motif the support of the conditionally stable motif.

Each stable motif of the Campbell et al. model can be interpreted as the smallest group of species that achieve and maintain a specific survival state. Thus, it can either be a sub-community (in which nodes have a stationary active state) or the simultaneous extinction of all species in the group (in which nodes have a stationary inactive state)^28^. Stabilization of each stable motif (i.e., achieving and then maintaining the associated state) restricts the system trajectories into a specific region of the state space called a trap space. A trap space is a subset of the state space that has two properties: (i) A subset of the nodes have a fixed state in the trap space and (ii) If the system enters the trap space, it cannot leave^36^.

Stable motifs and conditionally stable motifs supported by them stabilize sequentially and confine the system to the smallest possible region of the state space called minimal trap space. If all nodes are fixed to either their active or inactive state within the minimal trap space, it corresponds to a fixed point. One of the significant advantages of MPBNs is that in this framework attractors are the same as minimal trap spaces; a complex attractor in which a subset of nodes oscillate also corresponds to a minimal trap space. This minimal trap space contains the maximal possible number of nodes that can achieve a stationary state and is guaranteed to contain a single attractor^31^. Stable motif and trap space based attractor-finding algorithms do not rely on the full search of state space, hence can efficiently identify the attractors of Boolean models^37–40^.

### Logical domain of influence and stable motif driver sets

To study the magnitude of the effect of species extinction on plant–pollinator communities, we take advantage of the concept of Logical Domain of Influence (LDOI). LDOI determines the influence of a fixed node state or of a stabilized stable motif on the rest of the components in a system^41^. The LDOI of a node state $\sigma _i(t)=\sigma _i$, denoted LDOI$(\sigma _i)$, is determined by a percolation process on the expanded network (recall that each virtual node of the expanded network corresponds to the state of a node of the regulatory network). Starting from the virtual node that corresponds to $\sigma _i$, we follow the outgoing edges. There are two possibilities: (i) We reach another virtual node: in this case the edge represents a sufficiency relationship and the virtual node is added to the LDOI$(\sigma _i)$. (ii) We reach a composite node: in this case we only add the composite node to the LDOI$(\sigma _i)$ if all the virtual nodes that have incoming edges to the composite node are already in the LDOI$(\sigma _i)$. The LDOI of a set of node states (e.g., of a stable motif) can also be found. In this case the only difference is that we have multiple starting points in the percolation process on the expanded network. Fig. 1b illustrates this concept: the LDOI of the stable motif highlighted with dark green include the virtual nodes highlighted with light green, as well as the virtual nodes of the stable motif itself.

Another relevant concept implemented in this study is the stable motif driver set^42^. A driver set of a stable motif is a minimal set of node states (virtual nodes) whose LDOI contains all the node states in the stable motif. Each stable motif can have multiple driver sets. Driver sets can be internal, meaning that the stable motif contains the driver set, or external, meaning that the driver set resides outside the stable motif. The entire stable motif is contained in the LDOI of its driver set. Fig. 1b indicates one of the driver sets of each of the two stable motifs; the entire grey stable motif is in the LDOI of the virtual nodes {$\sim$po_1, $\sim$po_2, $\sim$po_3} outlined by solid black lines and the dark green stable motif is in the LDOI of {pl_2} outlined by solid black line. The LDOI algorithm is founded on the assumption that node states that correspond to the starting virtual nodes are fixed and maintained, and hence does not allow a node state contradictory to the starting virtual nodes to be added to the LDOI. The algorithm truncates the process at this point and does not move further in the expanded network. The python implementation of Ref.^41^ is available at https://github.com/yanggangthu/BooleanDOI.

### Conversion of Boolean threshold functions to Boolean logical functions

To construct the expanded network and perform stable motif analysis, the threshold functions need to be converted to Boolean logical functions using “AND”, “OR”, and “NOT” operators in disjunctive prime form. During this process, the species that do not receive any benefits from the species pool (i.e., the nodes that do not have any incoming positive edges) cannot establish. According to our analysis of the model, only 33.7% of the species pool have a chance to participate in a stable community. We omit the species that cannot participate in any stable community from the network, thus the effective network becomes smaller. As explained in detail in our previous work^28^, the conversion from threshold functions to Boolean disjunctive form also reveals negative edges that have no functional consequence. For example, having a positive regulator and up to three negative regulators is equivalent to just having the positive regulator: the target node will be active if the positive regulator is active and it will be inactive if the positive regulator is inactive. Such superfluous negative edges are deleted while the coupled positive edges remain in the network. We determined that among the species that have the opportunity to establish an average of 41.4% of the interactions are mutually beneficial, 36.7% are only beneficial in one way, and the rest are beneficial in one way and detrimental the other way. The persistence of some interactions with a negative impact on one of the species reflects the existence of interactions that are not true mutualisms in real ecological communities, including for instance nectar robbing.

In large networks with each node having numerous regulators, the converted Boolean functions become extensive due to combinatorial explosion. As an instance, for a node that has 3 positive regulators and 5 negative regulators the Boolean function in disjunctive prime form has 33 prime implicants, each involving an “AND” gate. Such a complex expanded network is very difficult and time-consuming to analyze. To tackle this problem, we previously developed a simplification method that eliminates randomly selected negative regulators for each node such that the remaining regulators retain the probability of $H(x)=1$^28^. This simplification method reduces the number of negative edges and simultaneously dials up the strength of the remaining negative edges such that the Boolean function is statistically equivalent to the original threshold function. Intuitively, the remaining negative regulators compensate for the dropped negative regulators and represent the collective cost of the wasted opportunities because of incompatible interactions. In the same example above, the simplified function only has 3 prime implicants, each corresponding to one of the positive regulators. This simplification method shifts the weight of the dropped negative regulators to the remaining ones and implicitly assumes that each remaining negative regulator can overcome the positive regulators; in other words, the collective cost of the negative regulators (including those dropped) can overcome the effect of active positive interactions. This assumption results in an inhibitor-dominant Boolean function. Assuming that node *i* has $N_p$ positive regulators and $N_n$ negative regulators, the simplified Boolean function in disjunctive prime form can be written as:

2 $$\begin{aligned} f_i= (\sigma _{P_1}(t) \text { OR}\dots \text {OR } \sigma _{P_{N_p}}(t)) \text { AND NOT }\sigma _{N_1}(t)\dots \text { AND NOT }\sigma _{N_x}(t) , \end{aligned}$$

in which $\{P_k\}$ are the positive regulators, $\{N_l\}$ are randomly sampled negative regulators, and *x* is the number of negative regulators we keep. The conservation of probability of $H(x)=1$ is used to calculate *x* (for more details see^28^). The conversion of the threshold functions to disjunctive prime form causes the deletion of 92.2% of the negative edges and our proposed simplification process results in the additional loss of 7.6% of the negative edges. Since these edges were always paired with positive edges with an opposite direction, plants and pollinators remain connected. As most of the simplified plant–pollinator networks do not include negative feedback loops, their expanded network is disjoint and its strongly connected subgraphs consist of only active or only inactive node states. The stable motifs consist of only active or inactive node states in such networks^28^.

### Network of functional relationships and identification of attractors

Following the conversion of the threshold functions to Boolean logical form, the construction of the expanded network is feasible and one is able to identify stable motifs and conditionally stable motifs supported by stable motifs. In our previous study^28^ we developed an efficient method to identify the conditionally stable motifs and their supports. Once the support(s) of each conditionally stable motif is (are) identified, a new entity called motif group is constructed; each motif group consists of the focal conditionally stable motif and one of its supports. Each of such motif group can stabilize independently of the rest of the network.

We also defined two functional relationships among stable motifs and motif groups. Two motifs or motif groups are mutually exclusive if they have at least one shared node that has different states in each of them. A motif is in the LDOI of another motif if the LDOI of the set of virtual nodes of the latter contains the virtual nodes of the former; this means the stabilization of the latter yields stabilization of the former. A usual case for an LDOI relationship between two stable motifs is if their virtual nodes overlap.

Constructing the network of functional relationships allows for identifying the maximal consistent combinations of stable motifs and motif groups; combinations that do not consist of mutually exclusive stable motifs and motif groups. Each of these consistent combinations then leads to one minimal trap space. The LDOI relationships were also implemented to speed up the process: Combinations of two stable motifs or motif groups that have a LDOI relationship do not need to be considered, as the effectiveness of that combination in trapping the system does not surpass the effectiveness of the source of the LDOI relationship.

Unlike the original stable motif analysis^37,38^, this method does not require exploring all sequences of stable motifs and conditionally stable motifs to identify minimal trap spaces; rather it relies on the functional relationships among stable motifs and conditionally stable motifs to identify the groups that lead to such trap spaces. As a result, it speeds up the attractor identification process^28^.

### Attractor control

An advantage of stable motif analysis is the insight it offers on the trajectories the system can take. For a vast majority of Boolean models and update methods (and in all MPBNs) the set of minimal trap spaces coincides with the set of attractors. The exceptions consist of so-called motif-avoidant attractors, which are complex attractors that visit the state space in such a way that avoids the activation of stable motifs available to the system^40^. The Campbell et al. model does not have motif-avoidant attractors^28^, thus in the following we will use “attractor” and “minimal trap space” interchangeably.

Each of the possible autonomous trajectories in the Campbell et al. model is characterized by the stabilization of a combination of stable motifs and leads to an attractor. As a result, each attractor can be uniquely identified by the corresponding combination of stable motifs. One can also use this information on the dynamical trajectories to drive the system toward a desired attractor by controlling each of the stable motifs in a sequence attractor^37,38^. To control a motif, one needs to identify and activate its driver set—a minimal set of node states that contains the whole stable motif in its LDOI^41,42^. Having the driver sets of the stable motifs in a sequence, the control set of each attractor (the set of nodes whose fixed state makes the system converge to the attractor from any initial state) can be identified. Typically there exist multiple control sets, and each contains a small number of node states^38,43^.

Stable motif-based control methods can also be implemented to drive the system away from an unwanted attractor. By blocking the stable motif (or family of overlapping stable motifs) that leads to the undesired attractor, it is impossible for the system to reach that attractor. For instance, Campbell et al. targeted specific interactions in the expanded network and destroyed them in order to prevent the formation of stable motifs that lead to an undesired attractor. They analyzed the expanded network and identified the smallest set of edges that if removed, the stable motifs that correspond to the undesired attractor and their stabilization effects are eliminated^44^. Stable motif based network control is specifically insightful in the study of extinction of species and restoration strategies in the case of plant–pollinator communities.

## Results

**Figure 2:**
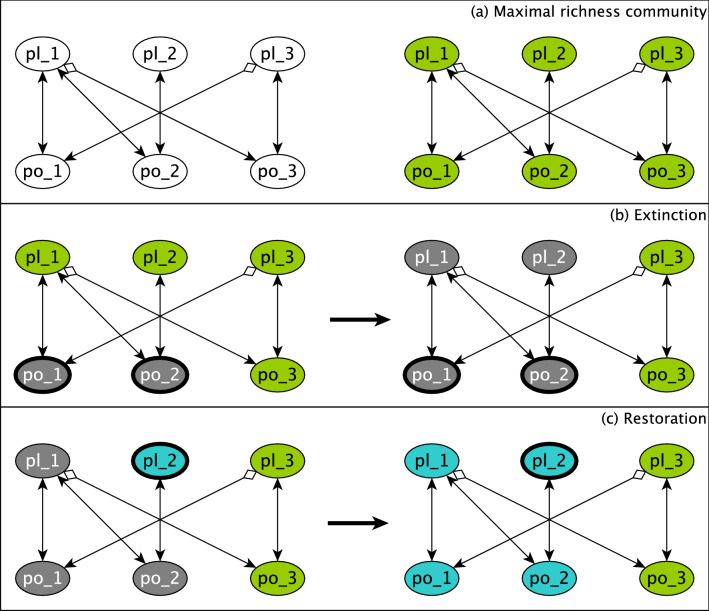
Illustration of species extinction and restoration in a hypothetical 6-species community. (**a**) The interaction network (on the left), and the maximal richness community possible for this network (the community with the most established species). Nodes highlighted with green represent established species. (**b**) The initial extinction of two species, po_1 and po_2 (left) and the community that results after cascading extinctions (right). Nodes highlighted with grey represent extinct species. (**c**) An intervention to restore pl_2 (left), which induces the restoration of further species, finally leading to a restored community with all the species present (right). The nodes highlighted with teal represent the restored species.

The Campbell et al. model provides an excellent framework to identify species whose extinction leads to community collapse and species whose reintroduction can restore the community (see Fig. 2 for an illustration of these processes). Our first objective, finding the effect of species extinction on the rest of the species in an established community, is achievable using the concept of Logical Domain of Influence (LDOI)^41^; the LDOI represents the influence of a (set of) fixed node state(s) on the rest of the components in a system. In this section we first present our proposed method to calculate the LDOI for the Boolean threshold functions governing the Campbell et al. model of plant–pollinator community assembly. Then we verify that the simplified logical functions preserve the LDOI and hence can be implemented to further analyze the effect of extinction in plant–pollinator networks. Next, we address one of the main questions that motivated this study: Can stable motif driver set analysis facilitate the identification of keystone species? We discuss the identification of the driver sets of inactive stable motifs and motif groups and present the results of stabilizing these sets to measure the magnitude of the effect of species extinction on the communities. Lastly we discuss possible prevention and mitigation measures based on the knowledge acquired from driver sets of stable motifs and motif groups.

### LDOI in the Boolean threshold model

**Figure 3:**
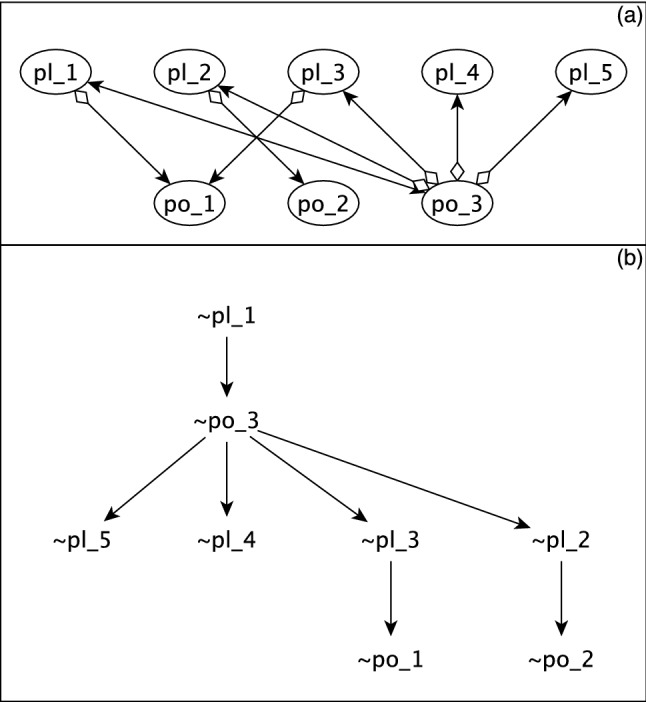
Breadth first search of the interaction network to find the LDOI of a (set of) fixed note state(s) in Boolean threshold functions governing the dynamics of plant–pollinator networks. (**a**) An interaction network with five plants and 3 pollinators. (**b**) The breadth first search in the case of starting from the node state $\sim$pl_1. The nodes with incoming edges from pl_1 make up $S_1=\{$po_1, po_3$\}$. The second sufficiency check is satisfied for node state $\sim$po_3, as a result $\sim$po_3$\in$ LDOI$(\sim$pl_1). The same process is applied for node po_1, but none of the sufficiency checks are satisfied, so this node will be visited again later. The next level of the search consists of the nodes that have incident edges from $S_1$, i.e., $S_2=\{$pl_2, pl_3, pl_4, pl_5$\}$. The second sufficiency check is satisfied for all of these nodes and they are all fixed to their inactive state in the LDOI$(\sim$pl_1). Lastly, we reach $S_3=\{$po_1, po_2$\}$. Node po_1 is reached again, and with both its positive regulators fixed to their inactive states the second sufficiency check is satisfied and node po_1 is fixed to its inactive state as well. The same holds for po_2 and hence LDOI$(\sim$pl_1$)=\{\sim$po_3, $\sim$pl_2, $\sim$pl_3, $\sim$pl_4, $\sim$pl_5, $\sim$po_1, $\sim$po_2 $\}$.

The LDOI concept was originally defined on Boolean functions expressed in a disjunctive prime form. Here we extend it to Boolean threshold functions. We implemented it as a breadth first search on the interaction network, as exemplified in Fig. 3. Assume that we want to find the LDOI of a (set of) node(s) $S_0=\{n_1,\dots ,n_N\}$ and their specific fixed state $Q(S_0)=\{\sigma _{n_1},\dots ,\sigma _{n_N}\}$. Starting from the set $S_0$, the next set of nodes $S_1$ that can acquire a fixed state due to the influence of $Q(S_0)$ consists of the nodes that have an incoming edge from the nodes in the set $S_0$ in the interaction network. The nodes in set $S_1$ are the subject of the first search level. For each node $n_i \in S_0$ and $n^\prime _i \in S_1$ we assume a “worst case scenario” (i.e., maximal opposition of the effect of $n_i$ on $n^\prime _i$ from other regulators) to find the possible sufficiency relationships between the two. There are five cases:

1. If $n_i$ is a positive regulator of $n^\prime _i$, then $\sigma _{n_i}=1$ is a candidate for being sufficient for $\sigma _{n^\prime _i}=1$. We assume that all other positive regulators of $n^\prime _i$ that have an unknown state (i.e., are not in $Q(S_0)$) are inactive and all negative regulators of $n^\prime _i$ that have an unknown state are active. If $\sum _j W_{ij}> 0$ under this assumption, then the active state of $n_i$ is sufficient to activate $n^\prime _i$. The virtual node $n^\prime _i$ that corresponds to $\sigma _{n^\prime _i}=1$ is added to LDOI($Q(S_0)$).
2. If $n_i$ is a positive regulator of $n^\prime _i$, then $\sigma _{n_i}=0$ is a candidate for being sufficient for $\sigma _{n^\prime _i}=0$. We assume all other positive regulators of $n^\prime _i$ that have an unknown state are active and all negative regulators of $n^\prime _i$ that have an unknown state are inactive. If $\sum _j W_{ij}\le 0$ under this assumption, then the inactive state of $n_i$ is sufficient to deactivate $n^\prime _i$. The virtual node $\sim n^\prime _i$ that corresponds to $\sigma _{n^\prime _i}=0$ is added to LDOI($Q(S_0)$).
3. If $n_i$ is a negative regulator of $n^\prime _i$, then $\sigma _{n_i}=1$ is a candidate for being sufficient for $\sigma _{n^\prime _i}=0$. We assume all positive regulators of $n^\prime _i$ that have an unknown state are active and all other negative regulators of $n^\prime _i$ that that have an unknown state are inactive. If $\sum _j W_{ij}\le 0$ under this assumption, then the active state of $n_i$ is sufficient to deactivate $n^\prime _i$. The virtual node $\sim n^\prime _i$ that corresponds to $\sigma _{n^\prime _i}=0$ is added to LDOI($Q(S_0)$).
4. If $n_i$ is a negative regulator of $n^\prime _i$, then $\sigma _{n_i}=0$ is a candidate for being sufficient for $\sigma _{n^\prime _i}=1$. We assume all positive regulators of $n^\prime _i$ that have an unknown state are inactive and all other negative regulators of $n^\prime _i$ that that have an unknown state are active. If $\sum _j W_{ij}> 0$ under this assumption, then the inactive state of $n_i$ is sufficient to activate $n^\prime _i$. The virtual node $n^\prime _i$ that corresponds to $\sigma _{n^\prime _i}=1$ is added to the LDOI($Q(S_0)$).
5. If none of the past four sufficiency checks are satisfied, the node $n^\prime _i$ will be visited again in the next search levels.

The second set of nodes that can be influenced, $S_2$, are the nodes that have an incoming edge from the nodes in the set $S_1$. The algorithm goes over these nodes in the second search level as described above. This search continues to all the levels of the search algorithm until all nodes are visited (possibly multiple times) and either acquire a fixed state and are added to the LDOI or their state will be left undetermined at the end of the algorithm. In Fig. 3, we illustrate this search to find the LDOI$(\sim$pl_1). The first search level is $S_1=\{$po_1, po_3$\}$; $\sim$pl_1 is sufficient to deactivate po_3, but not po_1. As a result, $\sim$po_3$\in$ LDOI$(\sim$pl_1). This process continues until all levels are visited and at the end of the algorithm LDOI$(\sim$pl_1$)=\{\sim$po_3, $\sim$pl_2, $\sim$pl_3, $\sim$pl_4, $\sim$pl_5, $\sim$po_1, $\sim$po_2 $\}$.

To measure the accuracy of the simplification method originally introduced in^28^, we analyzed logical domains of influence in 6000 networks with 50–70 nodes. These networks are among the largest in our ensembles and have the most complex structures. We randomly selected (sets of) inactive node states, found their LDOIs using the Boolean threshold functions and the simplified Boolean functions, and compared the two resulting LDOIs. We used 8 single node states and 8 combinations of size 2 to 4 for each network. We found that in all cases the LDOI calculated using the simplified Boolean functions matches the LDOI calculated using the Boolean threshold functions.

Next, we analyzed (sets of) active node states and their LDOIs in the same ensembles of networks. Similar to the previous analysis, we used 8 single node states and 8 combinations of size 2 to 4 for each network. Our analysis shows that in 77.1% of the cases the LDOI calculated using the simplified Boolean functions matches the LDOI calculated using the Boolean threshold functions. In 22% of the cases the LDOI calculated from the simplified Boolean functions contains the LDOI calculated from the threshold functions, and it also contains extra active node states, overestimating the LDOI by 57.5% on average. These additional members of the LDOI result from the fact that the simplified Boolean functions contain fewer negative regulators than the threshold functions. The guiding principle of the simplification method is that the probability of $H(x)=1$ conserves the probability of each node having an active state across all the states it can have. In contrast, the probability of the propagation of the active state is not necessarily preserved and tends to be higher in the simplified Boolean model; thus the LDOI of the active node states is overestimated in some cases.

In the rest of the cases (about 1%), the LDOI calculated from the simplified Boolean functions does not fully capture the LDOI calculated from the threshold functions. This again is caused by the sparsification of the negative edges in the simplified Boolean functions. In the threshold functions, the activation of 4 or more negative regulators of a target node combined with one active positive regulator is sufficient to deactivate the target node, i.e., there might be inactive node states in the LDOI of a set of active node states. However, some of these negative regulators drop in the simplified Boolean model and the inactive state of the target node is not necessarily in the LDOI of the set of active node states in the simplified case. This is the rare mechanism by which the simplified model might underestimate the influence of active node states on the rest of the network.

In the following section we are interested in analyzing the effect of species extinction on the established community, i.e., we look at the LDOI of (sets of) inactive node states. Observing that the influence of extinction of species is measured correctly in the simplified Boolean models, we conclude that these models can be utilized to further analyze the process of extinction and its ecological implications.

### Stable motif based identification of species whose loss leads to cascading extinctions

Each stable motif or motif group can have multiple driver sets; stabilization of each driver set leads to the stabilization of the whole motif or motif group. In plant–pollinator interaction networks, the stable motifs either represent a sub-community (when the constituent nodes stabilize in their active states) or the simultaneous extinction of all species in the group (when the constituent nodes stabilize to their inactive states). Stabilization of the nodes in the driver set of an inactive stable motif results in stabilization of all the nodes in the stable motif to their inactive state, i.e., cascading extinction of the constituent species.

The knowledge gained from stable motif analysis and the network of functional relationships offers insight into the cascading effect of an extinction that constitutes a driver set of an inactive stable motif. The magnitude of this effect depends on (i) the number of nodes that the inactive stable motif contains and (ii) the number of virtual nodes (including motifs and motif groups) corresponding to inactive species that are logically determined by the stabilization of the inactive stable motif.

To investigate the role of stable motifs in the study of species extinction in plant–pollinator networks, we simulated extinctions that drive inactive stable motifs in 6000 networks with the sizes of 50–70 nodes. We considered driver sets of size 1, 2, or 3, and implemented them by fixing the corresponding node(s) to its (their) inactive state. As a point of comparison, we also performed a “control” analysis using the same networks with the same size of initial extinction; however, the candidates of initial extinction are inactive node states that do not drive stable motifs or motif groups. Based on the properties of the drivers of stable motifs, one expects that following the extinction of driver species, cascading extinctions of other species follow, while the same does not necessarily hold for non-driver species. As a result, we expect to observe greater damage to the original community when driver species become extinct.

We assume that the “maximal richness community”—the community (attractor) in which the largest number of species managed to establish—is the subject of species extinction. This maximal richness community results from the stabilization of all active stable motifs. All other attractors that have some established species contain a subset of all active stable motifs and thus will contain a subset of the species of the maximal richness community. While for a generic Boolean model with multiple attractors one expects that a perturbed version of the model also has multiple attractors, this specific perturbation of a plant–pollinator model (namely, extinction of species in the maximal richness community) has a single attractor. We prove this by contradiction. Assume there are two separate attractors in the perturbed model, which means that there is at least one node that has opposite states in these two attractors. Note that this bi-stability is the result of the perturbation and not a property of the original system as the maximal richness community (an attractor) is the starting point for the introduced extinction. Specifically, the inactive state of the extinct node has to lead to the stabilization of another node to its active versus inactive states in the two separate attractors. The only case in which the stabilization of an inactive node state can result in the stabilization of an active node state is if there is a negative edge from the former to the latter in the interaction network after simplification. Since the Boolean function in 2 is inhibitor dominant, the negative regulators that remain in the Boolean model must be in their inactive states in the maximal richness attractor. As they are already inactive (extinct), they are not candidates for extinction. The only nodes that are candidates for extinction are the ones that positively regulate other nodes; perturbing the system by fixing these candidates to their inactive states cannot lead to the active state of a target node. In conclusion, bi-stability is not possible.

We found the new attractor of the system given the (combination of) inactive node state(s) using the the functions percolate_and_remove_constants() and trap_spaces() from the pyboolnet Python package. We quantify the effects of the initial extinction(s) on the maximal richness attractor by the percentage change in the number of active species, which we call damage percentage. Note that this choice of maximal richness community as the reference and starting point allows us to detect the cascading extinctions following the initial damage.

**Figure 4:**
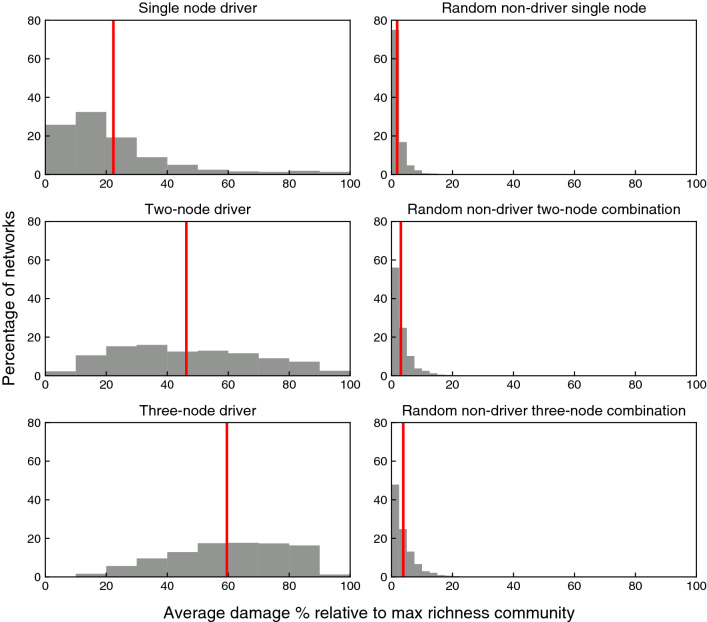
Histogram plots illustrating the average percentage of the damage caused in an established community after the extinction of species. This analysis is performed over 6000 networks with the size of 50–70 nodes. To study the extinction of species we started from the maximal richness community, then we fixed the nodes that correspond to the focal species to the their inactive states. The original extinctions are excluded from the damage percentages. The left column plots show the average damage percentage caused to the maximal richness community by the extinction of a driver set of size 1 (top), 2 (middle), or 3 (bottom) of an inactive stable motif or motif group. For each network, we determined all the relevant driver sets of one stable motif or motif group, we performed the extinction and calculated the resulting damage, then we calculated the average damage percentage over all data points collected for the same network. The right column plots show the average damage percentage caused to the maximal richness community by the extinction of 1 (top), 2 (middle), and 3 (bottom) non-driver, randomly chosen nodes. Each time a randomly selected combination of non-driver nodes were the subject of simultaneous extinction until all combinations are explored and then we calculated the average damage percentage over all data points collected for each network. The number of networks that qualify for each of these 6 categories differ (e.g., some networks have a stable motif with a driver set of size 2 but no stable motif with a driver set of size 3). In the left column 5529, 3212, and 1980 networks and in the right column 5779, 5626, and 5423 networks qualified respectively. The red lines represent the mean value of all the presented data points in each plot.

In Fig. 4 the left column plots show the average damage percentage caused by the extinction of 1 (top panel), 2 (middle panel), or 3 (bottom panel) species that represent driver sets of stable motifs and motif groups, while the right column plots illustrate the average damage percentage as a result of the extinction of 1, 2 or 3 species that represent non-driver nodes. Comparing the two columns, one can notice that stabilization of the driver sets of stable motifs and motif groups leads to considerably larger damage to the communities. This is due to the fact that stabilization of driver sets ensures the stabilization of entire inactive stable motifs and motif groups and hence ensures cascading extinctions. Comparing the plots in the left column, we see that the larger the driver sets are, the larger the damage to the community becomes. This is because larger driver sets are more likely to stabilize larger stable motifs and motif groups. This figure illustrates the significance of stable motifs and their driver sets in the study of species extinction in plant–pollinator communities.

**Figure 5:**
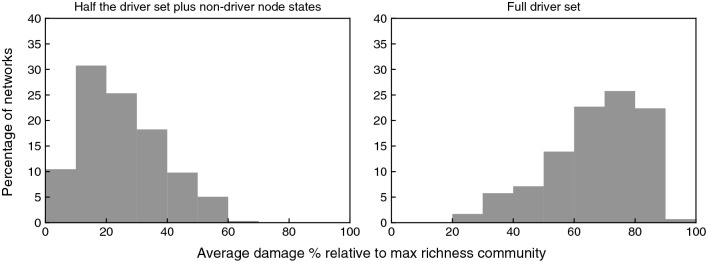
Histogram plots illustrating the average percentage of the damage caused in an established community after the extinction of species. This analysis is performed over 1000 networks with the size of 70 nodes (30 nodes representing plant species and 40 nodes representing pollinator species). The original extinctions are excluded from the damage percentages. The left plot shows the average damage percentage caused to the maximal richness community by the extinction of 2 species that are a subset of the 4-node driver set of an inactive stable motif or motif group plus 2 randomly selected non-driver species. The right plot shows the damage percentage caused to the maximal richness community by the extinction of 4-node driver sets of the same inactive stable motifs and motif groups. Each time the driver set of one stable motif or motif group was the subject of extinction and we calculated the average damage percentage over all data points collected for each network. 295 networks qualified for this analysis.

In Fig. 4 left column, the full driver set of one inactive stable motif or motif group was stabilized. However, the species that become extinct might only contain a subset of a driver set of a stable motif or motif group, i.e., they only stabilize a subset of the inactive node states in the stable motif or motif group. We compare the extinction effect caused by the stabilization of a full driver set of four nodes with the effect of the extinction of four nodes that contain a partial driver set in Fig. 5 using the batch of the largest networks in this study, i.e, the batch that contains networks with 30 nodes representing plant species and 40 nodes representing pollinator species. This choice is due to the fact that the existence of stable motifs and motif groups having a driver set of four node states is highly probable in larger networks. As expected, the stabilization of the complete driver set leads to greater damage. Stabilization of the same number of nodes that contain a partial driver set leads to significantly less damage and species loss in the community; the median damage percentage in the case of stabilization of partial driver sets is 22.6% while it is 69.2% in the case of stabilization of the full driver sets. We also note that damage of more than 90% occurs rarely and is only possible when a full driver set is stabilized (see Fig. 5 right plot). This suggests that the motif groups that lead to total extinction tend to have a driver set with more than four nodes; in other words, only the simultaneous extinction of five or more species would lead to total community collapse.

### Motif driver set analysis outperforms structural measures in identifying keystone species

**Figure 6:**
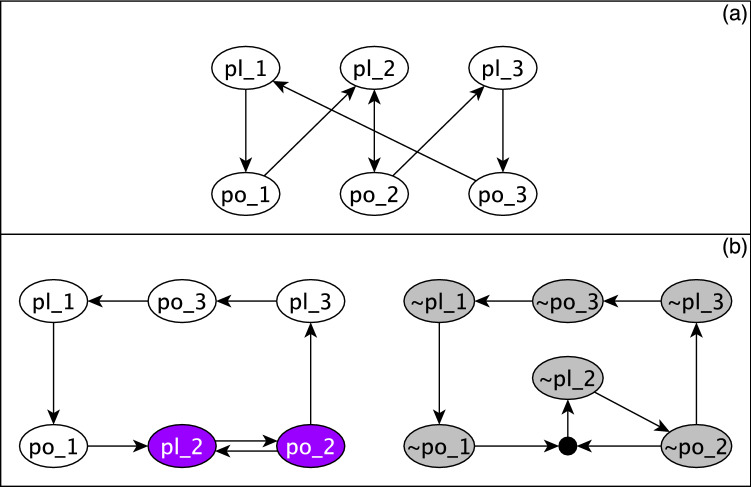
Generalist species in the interaction network and the expanded network. (**a**) A simplified network consisting of 3 plant and 3 pollinator species. pl_2 is a generalist species, i.e., it has two incoming edges indicating that it can survive on either of its sources of pollination, po_1 or po_2. The expanded network in (**b**) illustrates that the stabilization of the grey stable motif stabilizes all the nodes to their inactive states, and hence causes full community collapse. $\sim$pl_2 is the minimal driver set of the grey stable motif, consistent with the strong damage induced by the loss of a generalist species.

The literature on ecological networks offers multiple measures that reflect the importance of each species for community stability. One family of such measures is centrality (quantified by the network measures degree centrality and betweenness centrality). Previous studies^45,46^ have shown that species (nodes) with higher centrality scores are keystone species in ecological communities (i.e., species whose loss would dramatically change or even destroy the community). The nodes with highest in-degree centrality (such as pl_2 in Fig. 6a) represent generalist species that can receive beneficial interactions from multiple sources and survive. The nodes with highest betweenness centrality (such as pl_2 and po_2 in Fig. 6a) represent species that act as connectors and help the community survive. We find that high centrality corresponds to specific patterns in the expanded network: the inactive state of generalist or connector species is often the driver of a cascading extinction. Indeed, stable motif analysis of the expanded network in Fig. 6b confirms that there is an inactive stable motif (highlighted with grey) driven by the minimal set {$\sim$pl_2}. The fact that node pl_2 is a stable motif driver means that in the case of the extinction of pl_2 the whole community collapses.

To compare the effectiveness of stable motif analysis to the effectiveness of the more studied structural measures to identify keystone species, we performed an analysis similar to the previous section. We compared the magnitude of cascading extinctions in the case of extinction of stable motif driver nodes and of nodes with high values of previously introduced structural importance measures. Specifically, we used node betweenness centrality, node contribution to nestedness^47^, and mutualistic species rank (MusRank)^22^ to find crucial species based on their structural properties. For more details on definition and adaptation of these two measures see “Methods”. In this analysis, we used each measure to target species in the simplified Boolean models as follows:

1. Betweenness centrality: The 10% of species with the highest betweenness centrality are chosen to be candidates for extinction.
2. Node contribution to nestedness: The species with the most interactions tend to contribute the least to the community nestedness. Targeting them most likely leads to a faster community collapse^48^. As a result, 10% of species with the lowest contribution to network nestedness are chosen to be candidates for extinction. For more details on this measure, please see “Methods”.
3. Pollinator MusRank: The pollinator species with the highest MusRank importance are more likely to interact with multiple plants, so the 10% of pollinator species with the highest importance are chosen to be candidates for extinction. For more details on this measure, please see “Methods”.
4. Plant MusRank: The plant species with the highest MusRank importance are more likely to interact with multiple pollinators, so the 10% of plant species with the highest importance are chosen to be candidates for extinction.

**Figure 7:**
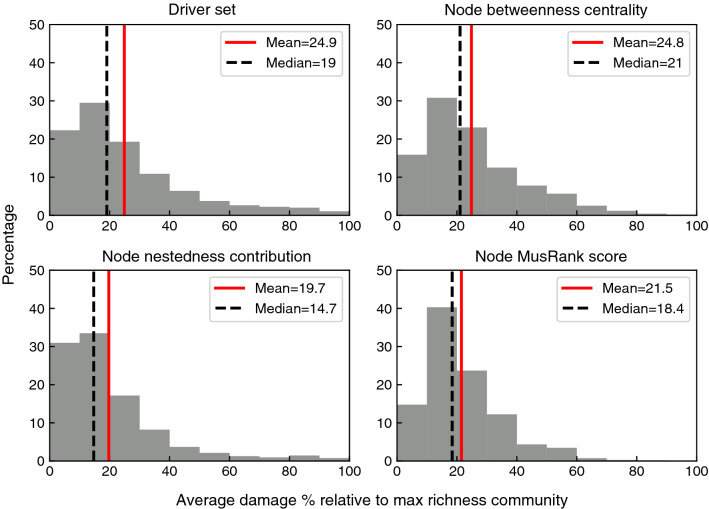
Histogram plots illustrating the performance of driver set analysis versus structural measures in identifying keystone species. The analysis was done on 6000 networks with sizes of 50–70 nodes. The starting point is the maximal richness community, i.e., the attractor in which the most species establish. For each network 1, 2, and 3 node(s) were selected and simultaneously fixed to their inactive states. After the cascading damage the new attractor is compared to the maximal richness attractor to calculate the damage percentage. The structural measures—betweenness centrality, node nestedness contribution, and node MusRank score—were calculated for all nodes in each network; the top 10% according to the relevant ordering were candidates to being fixed to their inactive states. The network IDs were matched, i.e., only the networks that had candidate nodes according to all four measures for each extinction size are included in this plot. The total number of data points is 6360. The red solid lines represent the mean and the black dashed lines represent the median over all data points in each plot.

Figure 7 illustrates the results of this analysis in 6000 networks with 50–70 nodes. In each network the 1-node, 2-node, and 3-node driver sets of inactive stable motifs are identified and made extinct. In the same networks 10% of nodes based on betweenness centrality, node contribution to nestedness, and node MusRank score were chosen to be candidates for extinction. To match the “driver set” data, all choices of 1, 2, or 3 nodes in these sets were explored and the damage was averaged over each extinction size for each network. We observe the cascading extinction and calculate the damage percentage relative to the maximal richness attractor. The plot represents the collective data over all initial simultaneous extinction sizes of 1, 2, and 3 species.

Comparing the four methods, one notices that the histograms acquired using stable motif driver sets, node betweenness centrality, and node contribution to nestedness are very similar, showing a peak for the 10–20% bin of the damage, and a long tail that reaches a damage percentage of 80–100%. The MusRank score performs less well in identifying the crucial species. Also, the frequency of the higher damage percentages shows that node contribution to nestedness is the closest to the “driver set” method in identifying nodes whose extinction causes the collapse of the whole community, making it the best structural measure out of the three. Nevertheless, the driver set method finds keystone species that cannot be identified via structural measures, as the corresponding damage percentage histogram has the most prominent tail at the right edge of the panel. Indeed, stable motif driver sets identified 82%, 80%, and 546% more species whose extinction leads to 60% or higher damage to the community when compared to betweenness centrality, node nestedness, and node MusRank score based methods respectively.

**Figure 8:**
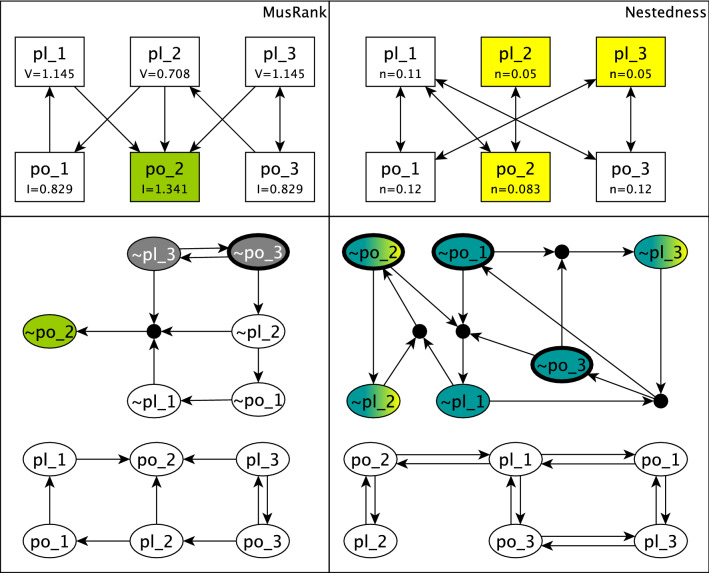
Networks illustrating examples of when structural measures fail to identify keystone species. In both columns simplified networks consisting of 3 plant and 3 pollinator species are presented. The MusRank is calculated for all the nodes in the network in the left column and denoted in the node labels. The expanded network corresponding to this network is shown below. Node contribution to network nestedness is calculated for all the nodes in the network in the right column and denoted in the node labels. Similarly the expanded network that correspond to it is shown below. Note that these two networks have different edges. In the left column MusRank score identifies node po_2, highlighted with green, as the most important, while the expanded network shows that the extinction of po_2 does not cause any further damage to the community, as this node has no outgoing edges. This is due to the fact that MusRank calculation process fails to consider the directed network and replaces all the directed edges with undirected ones. The MusRank score does not identify po_3 as a crucial species; however, virtual node $\sim$po_3, outlined with black in the expanded network is a driver of a stable motif that has all other nodes in its LDOI; the extinction of po_3 leads to full community collapse. In the right column, the nodes highlighted with yellow (pl_2, pl_3, and po_2) have the lowest node contribution to nestedness, which predicts that these nodes are likely crucial to the stability of the community. Analyzing the expanded network, one can see that these three nodes together are not able to drive the inactive stable motif highlighted with teal. The minimal driver set for this stable motif, outlined with black, consists of {$\sim$po_1, $\sim$po_2, $\sim$po_3}; together these nodes drive the inactive stable motif and cause full community collapse. The nestedness-based measure was not able to capture the significance of nodes po2 and po_3.

The reason for the higher effectiveness of driver set analysis is illustrated in Fig. 8 in which the MusRank score and node contribution to nestedness are calculated for two example networks. One can see how these two measures might incorrectly identify less vital species. In the left column of Fig. 8, MusRank identifies the node po_2, highlighted with green, as the most important species. However, this node does not have any outgoing edges; its extinction does not lead to any cascading extinction. The inability of the MusRank score to consider the direction of edges causes such misidentification. In the right column, the three nodes highlighted with yellow have the lowest contributions to network nestedness. The expanded network shows that these three nodes together are not able to cause full community collapse, while the three-node driver set of the inactive stable motif can. Since the nestedness definition depends on the number of mutual interactions, it might fail to identify some of the keystone nodes that are necessary to the stability of the community (for more details on node nestedness see “Methods”).

Previously it was shown that identifying the stable motifs and their driver sets can successfully steer the system toward a desired attractor or away from unwanted ones^37,38,43^. Stable motif analysis of the Boolean model offers insight into the dynamical trajectories of the system; hence control strategies can be developed accordingly. In the next section we use stable motif driver sets to suggest control methods and analyze their efficiency.

### Damage mitigation measures and strategies for endangered communities

There are two substantial questions related to managing the damage induced by species extinction: (1) How can one prevent the damage as much as possible? (2) Once the damage happens, the reintroduction of which species can restore the community and to what extent? In this section we aim to answer these questions in the context of the Campbell et al. model, implementing stable motif based network control. This analysis can inform agricultural and ecological strategies employed to prevent and mitigate damage.

#### Damage prevention

One of the most important questions in ecology is what strategies to use so that we can prevent and avert extinction damage to the community. In this section we analyze how the knowledge from stable motif analysis and driver sets can be implemented to minimize the effect of extinction of keystone species in case of limited resources. Each attractor of the original system can have multiple control sets; stabilizing the node states in each control set ensures that the system reaches that specific attractor. The same information from the attractor control sets can be implemented to prevent the system from converging into unwanted attractors. Zañudo et al. illustrated that by blocking (not allowing to stabilize) the stable motifs that lead to the unwanted attractors, one can decrease the probability (sometimes to zero) that the system arrives in those attractors^38^. In order to block an attractor, the control sets of that attractor are identified and the negations of the node states in the control sets are externally imposed. This approach eliminates the undesired attractor; however, new attractors might form that are similar to the eliminated attractor. Campbell et al. showed that in order to avoid such new attractors one needs to block the parent motif, which in this case is the largest strongly connected subgraph of the expanded network that contains the inactive virtual nodes^44^. Here, we investigate how stable motif blocking based attractor control can identify the species whose preservation would offer the highest benefit in avoiding catastrophic damage to the community. This information would aid the development of management strategies in plant–pollinator communities.

To avoid all attractors that lead to some degree of species extinction, one needs to block all the driver sets of all inactive stable motifs and motif groups in a given network. Implementing this in 100 randomly selected networks with 25 plant and 25 pollinator nodes, we found that 45.6% of the species in the maximal richness community need to be kept (prevented from extinction) to ensure the lack of cascading extinctions. Given that management resources are usually limited, active monitoring and conservation of almost half of the species in a community seems costly and impractical. Hence, we set a more feasible goal of identifying and blocking the driver set(s) of the largest inactive stable motif or motif group in each network. The same 100 networks containing 50 nodes are the subject of analysis in this section. The reason for performing the analysis in a relatively limited ensemble is that it involves the identification of all driver sets of the largest inactive stable motif or motif group, which is computationally expensive. For each network, the driver set of the largest inactive stable motif or motif group (which corresponds to the extinction of all the species in that group) is identified and blocked (that is, the corresponding species are not allowed to go extinct). Then the same number of species as in the driver set of that stable motif or motif group are selected and stabilized to their inactive state. We considered all combinations of node extinctions outside the blocked subset, calculated the damage percentage relative to the maximal richness community, and then averaged over all data points for each network. As a control, we repeated the analysis without blocking; the size of the initial extinction is the same as in the previous analysis for consistency.

**Figure 9:**
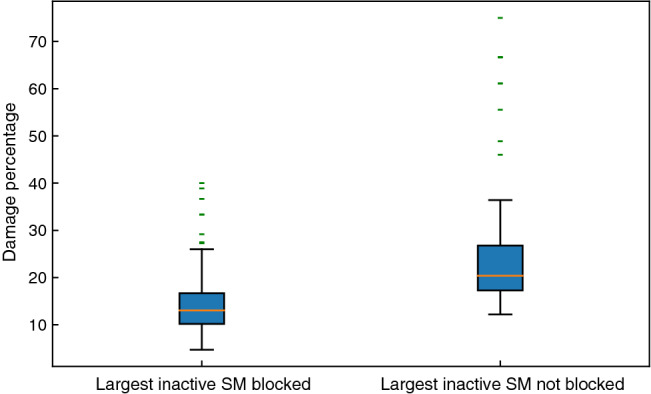
Box plots comparing the damage communities face if the largest inactive stable motif or motif group is completely blocked, i.e., all the drivers of this inactive stable motif or motif group are prevented from stabilizing versus if the same stable motif or motif group is allowed to stabilize. This analysis was performed over 100 randomly selected networks that contain 25 plant and 25 pollinator nodes. All the driver sets of an inactive stable motif or motif group are identified. From left to right the box and whiskers plots show the average damage percentage relative to the maximal richness community if the largest inactive stable motif is blocked and the same quantity if the largest stable motif or motif group is not blocked respectively. For the left box and whiskers plot, all combinations of inactive node states except the driver sets are considered, and for the right box and whiskers plot all combinations are explored. Due to the computational complexity caused by combinatorial explosion, this analysis was performed over 100 randomly selected 50-node networks.

Figure 9 shows the result of the analysis described above for 100 networks. The left box and whiskers plot illustrates the damage percentage relative to the maximal richness community when the blocking feature is activated, while the right box and whiskers plot shows the damage percentage relative to the maximal richness community when the blocking is disabled. The average and median damage percentages are 14.96% and 13.04% respectively when the largest inactive stable motif or motif group was blocked and 24.73% and 20.38% when it was not. This $\sim$10% difference in the average between the two sets of results, as well as the fewer cases of high-damage outliers in the left plot, demonstrates that by preventing the extinction of species identified by stable motif analysis, one can prevent catastrophic community damage considerably.

To estimate the fraction of species that would need to be monitored to prevent their extinction, we compared the size of the maximal richness attractor and the size of the driver set of the largest stable motif. The maximal richness community represents an average of 32% of the original species pool, approximately 15 out of 50 species. The driver sets of the largest stable motifs had an average size of 2.5 node states over all 100 networks, i.e., about 16.6% of the maximal richness community. In ecological terms, given limited resources, the information gained from stable motif driver sets can help direct the conservation efforts toward the keystone species that play a key role in maintaining the rest of the community in a cost-effective manner.

#### Restoration of a group of species

Although human preservation efforts have been directed toward community conservation, there are many industrial activities that lead to ecosystem degradation. Ecologists are interested in developing restoration strategies to be deployed after a stable community is hit by catastrophic damage to recover biodiversity and the ecosystem functions it provides^49^. Here we propose that stable motif analysis and the driver sets identified from the expanded network can give insight into restoration measures. While we examined the inactive stable motifs in the study of species extinction, here we focus on the active stable motifs as our goal is to restore as much biodiversity as possible.

Several network measures have been proposed to identify the species that if re-introduced would restore the community considerably. Two of the most studied algorithms include maximising functional complementarity (or diversity) and maximising functional redundancy^50^. The first strategy targets the restoration of the species that provide as many functions to the ecosystem as possible; this approach results in a community that has a maximal number of functions provided by different groups of species. Alternatively, maximising the functional redundancy yields a community in which several species perform the same function. While this resultant community might have a limited number of functions, it is robust. Both of these community restoration approaches have been studied extensively (e.g. see^21^).

We hypothesize that restoring the species that constitute driver sets of active stable motifs can help maximise the number of species post-restoration. Since there is evidence that functional diversity correlates with the number of species in the community^51^, we compare the post-restoration communities identified by stable motif driving with the functional diversity maximisation approach. As discussed in section *LDOI in the Boolean threshold model*, the Boolean simplification of the threshold functions leads to an overestimation of the LDOI of active node states (compared to the original threshold functions) in some networks. We evaluate the negative effects of this overestimation by checking the effectiveness of the restored species in the original threshold model.

The same 6000 networks we examined in the last section were the subject of this analysis. To create an unbiased initial community, we create the damaged communities by eliminating the same number of species from the maximal richness community as the number that will be restored. We identify the inactive stable motif or motif group with the driver set size of 1, 2, or 3 node states that causes the most damage to the maximal richness community. We then eliminate the species corresponding to this driver set to reach the most damaged community for the given size of the initial extinction. This community is the starting point for two analyses. In the stable motif driving approach we stabilized an active stable motif that has a driver set of the same size as the initial extinction to reach a post-restoration community and calculated the percentage of the extinct species that were restored. In the functional diversity maximization based approach we re-introduced the same number of species selected from the to 10% of species in terms of their contribution to functional diversity.

To calculate the functional diversity of a community one needs to (1) define and construct a trait matrix, (2) determine the distance (trait dissimilarity) of pairs of species, (3) perform hierarchical clustering based on the distances to create a dendrogram, and (4) calculate the total branch length of the dendrogram, i.e., the sum of the length of all paths^51,52^. Petchey et al. argued that resource-use traits among plant and pollinator species can be used to classify the organisms into separate functional groups^53^ and Devoto et al. proposed the use of the adjacency matrix based on the interaction network as the trait matrix^21^. In this study we do the same and implement the bipartite adjacency matrix to construct the distance matrix.

Since the networks of the Campbell et al. model are directed, we modify the algorithm in that we have two separate adjacency matrices, one denoting the edges incoming to plant species and the other denoting the edges incoming to pollinator species. The hierarchical clustering algorithm is then run on each of these matrices separately, resulting in a dendrogram for each adjacency matrix. If extinction occurs in a community, the functional diversity of the survived community can be determined by calculating the total branch length of the subset of the dendrogram that includes only the survived species. The restoration strategy using this method is to re-introduce the nodes whose branches add the most to the total branch length of this subset, i.e., maximise the functional diversity of the survived community^54^. For more details see “Methods”.

**Figure 10:**
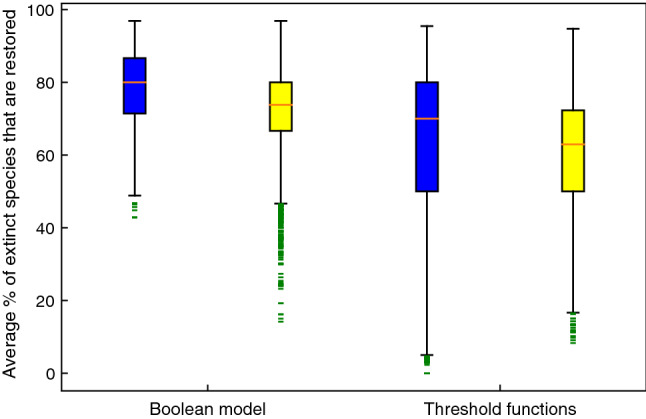
Box and whiskers plots illustrating the average percentage of the extinct species that are restored following the stable motif driver set restoration strategy (blue) versus the functional diversity based approach (yellow). This analysis is performed over 6000 networks with sizes of 50–70 nodes. Starting from the maximal richness community, for each network one inactive stable motif with a driver set of 1, 2 or 3 nodes was stabilized to reach a new damaged community. This task was performed until the community with the most extinct species was identified. This is the community we set as the starting point for the restoration process using both methods. The pair on the left represents the two methods applied to the simplified Boolean model. For both methods we identified 1, 2, or 3 influential nodes for community restoration and we calculated the percentage of the extinct species that could be restored. The pair on the right represents restoring the same species identified by each method in the previous analysis in the original threshold model. In all analyses the community restoration percentage was averaged over all combinations of the same size, for each network and each method. The IDs of all networks are matched.

In each network, the percentage of the extinct species that were restored was calculated and averaged over all data points for each restoration size and each network. Figure 10 illustrates the results of this investigation. Applied to the simplified Boolean model, the median restoration percentage in the case of active stable motif driver set method (blue plot) is 80%. The functional diversity maximization strategy to restoration (yellow plot) yields a lower median restoration percentage, 73%, as well as a large number of low-restoration outliers. Although one might argue that identifying beneficial species using the functional diversity maximization strategy works well, the higher percentage of the cases of 80–100% restoration in case of the active stable motif driver set analysis indicates that the latter identifies some of the most effective restorative species that are not identified via the former method. As in a minority of cases the simplified Boolean model overestimates the positive impact of the sustained presence of a species (see section *LDOI in the Boolean threshold model*), we sought to verify the effectiveness of the predicted restoration candidates in the original threshold model. The blue (respectively, yellow) box and whiskers plot on the right represents the restoration percentages of the same species as in the left blue (respectively, yellow) plot when these species are restored in the threshold model. The median of the right blue plot is 70%, while the median of the right yellow is 63%, preserving the advantage of the stable motif driver sets. We conclude that although the simplified Boolean model overestimates the restoration effectiveness of certain driver sets (visible in the fact that the lower whisker of the blue plot on the right goes well below the lower whisker of the blue plot on the left), stable motif driver sets are more effective in both comparisons.

#### Community restoration via attractor control

As illustrated in section “Restoration of a group of species”, stable motif analysis identifies promising and cost-effective group restoration strategies. In this section we aim to go further and identify interventions that can maximally restore a community. Previous stable motif based network control methods^37,38,55^ require a search for the smallest set of node states to control the system once the stable motif stabilization trajectories are identified. This smallest set may not contain a node from each stable motif in the sequence. In this work, however, we know that each stable motif or motif group needs to be controlled individually^28^ because the stabilization of none of the motifs results in the stabilization of another. As a result, the control set of each attractor is the same as the union of the driver sets of all members in the consistent combination corresponding to that attractor.

In this section we examined this attractor control method by setting the communities with 70% or more of the species in the maximal richness community as the target, i.e., the attractors that have 70% of the species in the maximal richness community are assumed to be the desired attractors. We then recorded the size of the minimal control set needed to achieve each of these attractors. Note that stabilizing each of these control sets guarantees that the system reaches the corresponding attractor^38^.

**Figure 11:**
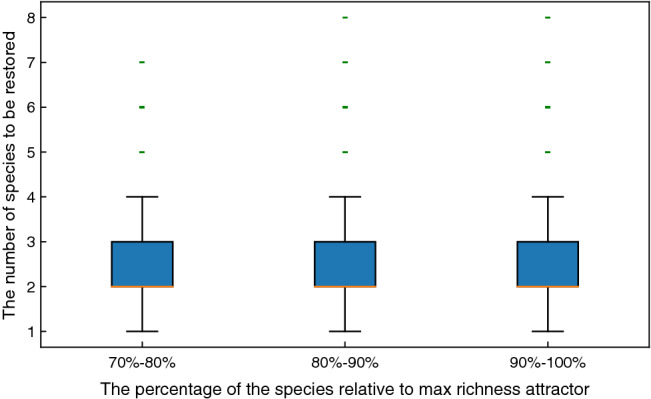
The number of species that need to be restored to save 70% of more of the species in the maximal richness community. In this analysis 6000 networks with 50–70 nodes were the subject. For each networks all the attractors that have 70% or more of the species in the maximal richness attractor are identified and set to be the target attractors. The control set of these attractors are then classified into three groups based on the percentage as illustrated in the figure. From left to right, the box and whiskers represent the size of the control set of attractors that have 70–80%, 80–90%, and 90–100% of the species in the maximal richness attractor respectively.

For this section, we analyzed 6000 networks that have 50–70 nodes. Figure 11 represents box-and-whiskers plots of the size of the minimal set of species that need to be restored, where the target community sizes are classified into three groups based on the percentage of the species relative to the maximal richness attractor. One can see that in half of the cases, the restoration of either 1 or 2 species manages to restore more than 70% of the maximal richness community. The largest set has 8 species that need to be restored; however, this data point is an outlier. As illustrated, driver set analysis and stable motif based attractor control can efficiently identify the species that play an influential restorative role and suggest management strategies that are effective at the scale of the whole community. To assess the impact of the LDOI inflation on this result, we used the restoration candidates identified by control sets of the attractors of the Boolean model in the threshold functions of a subset of networks. The results of comparing the restoration percentage is shown in Fig. 14. The first quartile, median and third quartile values are 78.26%, 86.6%, and 100% for the simplified Boolean models and 43.78%, 72.41%, and 85.71% for the threshold model.

To further compare the results of restoration obtained from the two models we sorted the species in the order of their contribution to community restoration following a catastrophic damage. We randomly selected 100 of the largest (70-node) networks, which have the highest probability of a discrepancy between the threshold functions and the simplified Boolean model. In 72% of the cases the two rankings matched completely, and in the majority of the remaining cases only one species was misplaced in the simplified Boolean model-based ranking. To conclude, there is a significant advantage to the implementation of the simplified Boolean model and the drawback can be addressed by a follow-up checking on the original threshold functions.

## Discussion

Network-based modeling of ecological systems such as plant–pollinator communities holds significant value as it captures the behavior of such systems without needing fine-grain details; this analysis has contributed useful insights to maintaining species^56^. In this work we specifically focused on the study of extinction and restoration of species and communities in plant–pollinator interaction networks and analyzed the role of generalized positive feedback loops in developing an understanding of the behavior of the system in the case of perturbation. To achieve this, we utilized the dynamic Boolean network model of mutualistic plant–pollinator community assembly developed by Campbell et al.^23^. This model is well-studied and its results have been supported by experimental evidence; for example, the potentially beneficial effect of the introduction of a generalist plant into an existing plant–pollinator community has been experimentally verified by Russo et al.^57^.

The analysis in this work relies on the concept of expanded network^32,33^ and its construction. The expanded network representation integrates the dynamic model and the structure of the interaction network. The benefit of such representation is that one can identify the so-called stable motifs—the smallest group of nodes that can maintain their state regardless of the state of the rest of the nodes in the network. To achieve this, in our previous work we converted the Boolean threshold functions to disjunctive prime logical form by consolidating negative edges^28^. Here, we showed that the logical domain of influence (LDOI) of the inactive node states predicted by the latter matches the LDOI calculated by the former, concluding that the disjunctive prime form, although simplified, is a suitable approximation for the Boolean threshold functions when studying species extinction.

While the same does not hold for the LDOI of the active node states, we showed that in the majority of the analyzed cases the LDOI calculated using the simplified Boolean functions matches the LDOI calculated from the threshold functions. In 22% of the analyzed cases, the simplified Boolean functions overestimated the LDOI of the active node states. To assess the quantitative effect of this negative impact, we calculated the percentage of community restoration on the original threshold models when restoring the species indicated by the stable motif driver sets and Functional Diversity maximization method, respectively, on the simplified models. We found that both methods are affected by this inflation in the LDOI of the active node states. Nevertheless, the restoration candidates identified by the stable motif driver set method outperform the candidates identified by the functional diversity maximization method. The additional states in the LDOI of an active node state may lead to an overestimation of the effectiveness of certain restoration candidates. We suggest that during the selection of restoration strategies the user/modeler use the stable motif driver sets on the simplified Boolean to identify the restoration candidates and then determine the LDOI of these candidates on the threshold functions using the algorithm described in the first section of the Results. As If the LDOI demonstrates that the target outcome can be achieved via the restoration of the focal node(s), one can move forward with the identified restoration strategy. Any misidentified node(s) can be disregarded. As there are multiple driver sets, and thus multiple predicted restoration candidates, we expect that a sufficient number of effective restoration strategies remain.

Previously we illustrated that identification of subgraphs and their relationships simplifies the task of attractor identification^28^. In this study, we build on this method to identify the attractor control sets—sets of node states that if stabilized, the system is guaranteed to settle in the corresponding attractor. Stable-motif based attractor control is particularly useful when studying species extinction and community restoration. We attempted to answer specific questions regarding the sustainability of plant–pollinator communities by identifying the generalized positive feedback loops in the corresponding interaction networks. Our results indicate that the analysis of positive feedback loops shows promising potential to be used as a complementary tool in community management endeavours. Specifically, we used the positive feedback loops to identify three groups of species: (1) keystone species whose loss lead to considerable damage to the community, (2) the subset of keystone species that should be prioritized for conservation efforts given the limited management resources, and (3) species whose restoration benefit the community the most.

While the valuable information given by the driver sets depends on the construction of a Boolean model as an added layer to the bipartite network, the authors believe that the intuitive nature of the Boolean modeling framework proposed by Campbell et al. study^23^ allows for the construction and parameterization of Boolean models of other mutualistic networks. The Campbell et al. model entails an ensemble of synthetic plant–pollinator networks whose properties reflect the statistical properties of real plant–pollinator networks. To be able to quantify the size of the effect of species extinction or restoration from a Boolean model of a specific mutualistic network, the model needs to incorporate the actual species as nodes, the actual interactions as edges, the actual benefits of each interaction (e.g., arising from the morphological match between species). The full complement of information is not generally available, especially for large networks, and therefore cannot at present be systematically incorporated into our analysis. Without a quantitative match between the real system and model, a quantitative match between effect sizes cannot be expected. Instead, the Campbell et al. synthetic model and our analysis explain the qualitative behavior of the community (the gain or loss in species composition) in the case of extinction and re-introduction of species. Our work establishes a general and efficient method to identify keystone species and the species whose restoration benefits the community considerably. The methodology of this study can be straightforwardly implemented for real mutualistic communities once the aforementioned information becomes available.

## Methods

### Identification of stable motif driver sets

Each stable motif has a driver set—a minimal set of node states that contains the whole stable motif in its LDOI. Previously Yang et al.^41^ and Rozum et al.^43,55^ developed python packages that include functions that identify the driver sets of stable motifs through brute-force evaluation. In this study, we implemented the idea that in order for a strongly connected component of the expanded network to be composite-closed, the virtual nodes that are targets of composite nodes within that component should be reachable. We optimized the process of finding the driver sets by taking advantage of a natural separation of virtual nodes based on their position relative to composite nodes (AND gates). A virtual node that has outgoing edges to only composite nodes cannot be a driver, as it is not sufficient for the stabilization of any other virtual nodes. In contrast, a virtual node that is a target of an edge starting from a composite node is much more likely to be a in a driver set; one can start the search for a driver set by starting from the virtual nodes that have an incoming edge from the composite nodes within that stable motif. This implementation simplifies the task of identifying the driver sets and reduces the search time as we assign a priority to the virtual nodes with an incoming edge from the composite nodes instead of brute-force evaluation. As an example one notices that all the virtual nodes that are in one of the driver sets of the grey stable motif in Fig. 1 are nodes that have incoming edges from the composite nodes within the same stable motif.

### Measures to identify keystone species

#### Node contribution to nestedness

A perfectly nested network is defined as a network in which for any two arbitrary nodes *i* and *j*, if the degree of *i* is smaller that the degree of *j*, then the vicinity (the set of neighbors) of *i* is contained in the vicinity of *j*^58^. Intuitively, nestedness characterizes the propensity that each node’s edges can be categorized as subsets of another node’s edges. It has been shown that ecological networks are highly nested^59^, i.e., the species with which the specialists interact are a subset of the species with which generalists do. Bascompte et al. have shown that empirical mutualistic networks (e.g., networks of plants and pollinators) are highly nested^59^ and introduced a measure based on the number of shared partners to quantify nestedness in such networks. Assume *M* is the adjacency matrix of an undirected network, in which $M_{ij}$ is 1 if there is an interaction between nodes *i* and *j* and 0 otherwise. Each node in this network is characterized by a degree $k_i=\sum _jM_{ij}$. Based on Bastolla et al.’s findings^9^, species *i* and *j* benefit if their number of shared symbiotic neighbours, $n_{ij}=\sum _{l}a_{il}a_{lj}=(M^2)_{ij}$ is as large as possible. As a result, Jonhson et al. propose to use

$$\begin{aligned} \eta _i=\frac{1}{N}\sum _j\frac{n_{ij}}{k_ik_j}=\frac{1}{N}\sum _j\frac{(M^2)_{ij}}{k_ik_j} \end{aligned}$$

as the contribution of node *i* to the network nestedness^47^. This study is focused on undirected mutualistic networks, while our networks are directed. We adapted the measure above as follows

3 $$\begin{aligned} \eta _i=\frac{1}{N}\sum _j\frac{n_{ij}}{k^{in}_ik^{in}_j}=\frac{1}{N}\sum _j\frac{(M_d^2)_{ij}}{k^{in}_ik^{in}_j} \end{aligned}$$

in which $M_d$ is the adjacency matrix of the directed network and $k^{in}_i$ and $k^{in}_j$ are the in-degree of nodes *i* and *j* respectively. The choice of in-degree reflects the properties of generalist species that have many incoming interactions and can survive under perturbations. According to Eq. (3), the species that have a large fraction of shared neighbours and a low in-degree have a large contribution to network nestedness. These species have a low number of interactions, but those are mutual with other species from the same group. Such species with the most contribution to community nestedness are the most vulnerable ones to extinction^48^, i.e, they are susceptible to extinction if their limited sources vanish from the community. On the other hand, the species with few mutual partners and a large in-degree contribute less to network nestedness. They are more resilient to extinction as they have multiple sources of benefit.

#### Mutualistic Species Rank (MusRank)

The novel non-linear ranking algorithm called Mutualistic Species Rank (MusRank) was proposed to identify the crucial species whose extinction causes considerable damage to a mutualistic community^22^. In this work, plants are considered to be the passive species and pollinators are the active species. Their rankings are named Vulnerability for the passive and Importance for the active species. It is assumed that the importance of an active species is the sum of the vulnerabilities of its passive partners; the more passive partners an active species has and the more vulnerable they are, the more important the active species is. The vulnerability of a passive species, on the other hand, is bounded by the least important active neighbours it interacts with. The vulnerability of passive species $V_P=1,\dots ,P_{max}$ and the importance of active species $I_A=1,\dots ,A_{max}$ are calculated at iteration *n* as a function of their value at iteration $n-1$ as follows:

4 $$\begin{aligned} \begin{aligned} \tilde{I}_A^{(n)}&=\sum _{P=1}^{P_{max}}M_{AP}V_P^{(n-1)} \rightarrow I_A^{(n)}=\frac{\tilde{I}_A^{(n)}}{\bigg \langle \tilde{I}_A^{(n)}\bigg \rangle _{A}}, \\ \tilde{V}_P^{(n)}&=\frac{1}{\sum _{A=1}^{A_{max}}M_{AP}\frac{1}{I_A^{(n-1)}}} \rightarrow V_P^{(n)}=\frac{\tilde{V}_P^{(n)}}{\bigg \langle \tilde{V}_P^{(n)}\bigg \rangle _{P}}. \end{aligned} \end{aligned}$$

For each vulnerability and importance, first the intermediate value is calculated on the left and then normalized to the mean value on the right. This iterative calculation continues until it reaches a fixed point in which a ranking of importances and vulnerabilities is achieved. This study found that the fixed point does not depend on the initial conditions, hence we chose the value of 1 for all importances and vulnerabilities at iteration $n=0$.

### Maximisation of functional diversity as a restoration strategy

This approach relies on hierarchical clustering, which is an unsupervised machine learning technique. To calculate functional diversity of a community we use the bipartite adjacency matrix in place of a trait matrix. We used the Euclidean distance between two columns of the adjacency matrix as the dissimilarity measure of the corresponding two species. We perform hierarchical clustering based on the distances to create a dendrogram. Specifically, we implemented a nearest point clustering algorithm using the function cluster.hierarchy.linkage() from the python package scipy. We then calculate the total branch length of the dendrogram, i.e., the sum of the length of all paths^51,52^. If extinction occurs in a community, the functional diversity of the survived community can be determined by calculating the total branch length of the subset of the dendrogram that includes only the survived species. To develop a restoration strategy using this method, one needs to re-introduce the nodes whose branches add the most to the total branch length of this subset, i.e., maximise the functional diversity of the survived community^54^.

**Figure 12:**
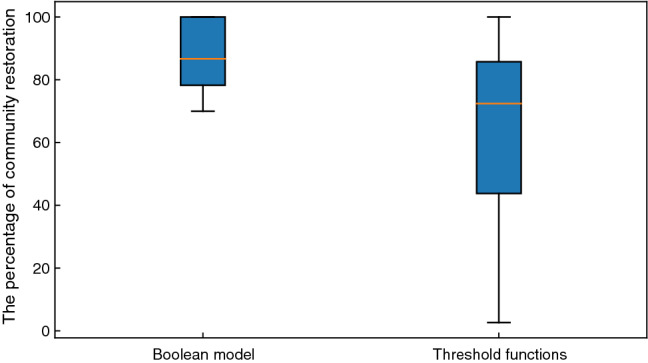
Box and whiskers plots illustrating the percentage of the community restoration following the restoration of the candidates identified by attractor control sets of the Boolean model. The method described in section Community restoration via attractor control was used in 1000 networks with 35 plant nodes and 25 pollinator nodes. The species identified by the control sets of the attractors that represent at least 70% of the maximal richness community were restored in the simplified Boolean model (the plot on the left) and in the original threshold functions (the plot on the right). The first quartile, median, and third quartile values are 78.26%, 86.6%, and 100% for the left box and whiskers, and 43.78%, 72.41%, and 85.71% for the right box and whiskers respectively.

**Figure 13:**
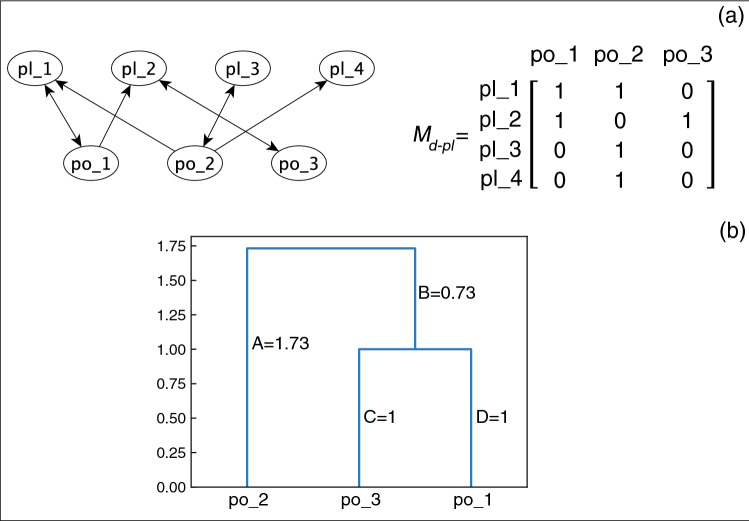
Calculation of functional diversity for a subset of the community. (**a**) Illustrates an interaction network with 4 plant and 3 pollinator nodes and the directed adjacency matrix $M_{d-pl}$ containing the binary information on the edges incoming to plant nodes. (**b**) Shows the dendrogram resulted when the matrix $M_{d-pl}$ was used for clustering. There are four branches labeled with letters A through D. The functional diversity of any subset of the dendrogram is equal to the sum of the branch lengths (considering the branches from the base of the dendrogram) of that subset. For example, the branch length corresponding to po_3 is the sum of the lengths of branches B and C, i.e. 0.73+1.

**Figure 14:**
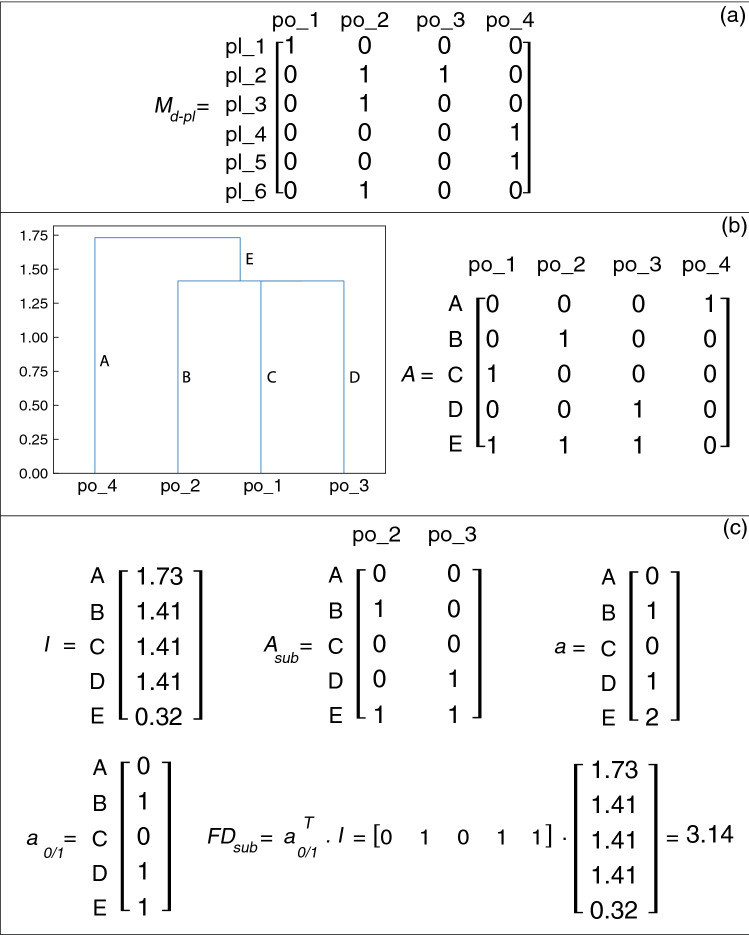
An example illustrating the calculation of functional diversity for a subset of a community for a network that consists of 25 plant and 25 pollinator nodes using linear algebra. This network reduces to 6 plant and 4 pollinator nodes after simplification. (**a**) Demonstrates the directed adjacency matrix $M_{d-pl}$ containing the binary information on the edges incoming to plant nodes. (**b**) Shows the dendrogram resulted when the matrix $M_{d-pl}$ was used for clustering. There are five branches labeled with letters A through E. The functional diversity of any subset of the dendrogram is equal to the sum of the branch lengths (considering the branches from the base of the dendrogram) of that subset. For example, the branch length corresponding to po_1 is the sum of the lengths of branches C and E. To calculate functional diversity of a sub community using algebra, first we calculate the distribution matrix, which denotes the distribution of branches in each pollinator. For instance, po_1 goes through branches C and E to reach the base of the dendrogram. As a result, rows C and E are equal to 1 under column po_1. (**c**) Illustrates the last final steps to cal; vector *I* denotes the length of each branch, and matrix $A_{sub}$ denotes the sub-matrix of *A* that only involves po_2 and po_3 (assume an example in which only po_2 and po_3 survive among pollinators). Then vector *a* which is the sum of the columns of $A_{sub}$ is constructed and converted to a binary vector $a_{0/1}$. Functional diversity of the survived community is then equal to the transposed $a_{0/1}$ multiplied by the vector *I*.

Figure 12a shows an interaction network made up of 4 plant and 3 pollinator nodes. The adjacency matrix that contains the information about the edges incoming to plant nodes is denoted by $M_{d-pl}$ and shown within the same panel. The matrix $M_{d-pl}$ is fed as the trait matrix to the function linkage() to generate the clustering data and dendrogram in Fig. 12b. This function calculates the Euclidean distances between the two columns of the adjacency matrix that corresponds to a pair of species. For instance, the distance between po_1 and po_2 is $\sqrt{((1-1)^2+(1-0)^2+(0-1)^2+(0-1)^2}=1.73$ and the distance between po_1 and po_3 is 1. Based on the distances, the function clusters the nodes (in this case pollinator nodes); nodes (or clusters) with the smallest distance are clustered together at each step until no node remains. In the resulting dendrogram the vertical segments indicate the Euclidean distances between the nodes. The functional diversity of the whole community equals the sum of the branch lengths of the dendrogram, i.e., functional diversity is 4.46 in this case. Let’s assume among pollinators only po_3 survives the extinction; in this case functional diversity of the community is equal to the branch length of po_3, 1.73. To choose a restoration strategy using this method, one needs to re-introduce the nodes that increase functional diversity the most. For instance, in this example let’s assume we can only restore one pollinator. The addition of po_1 adds 1 to the functional diversity, while the addition of po_2 adds 1.73 according to the dendrogram. In this case, the restoration priority belongs to po_2. Looking at the interaction network in panel (a), one realizes the significance of po_2 over po_1 as po_2 benefits pl_4 that no other pollinator can save. The same algorithm was also performed to cluster plant nodes and identify restoration priorities among them. Fig. 13 demonstrates how functional diversity can be calculated using linear algebra in the context of a more complex example network. The functions that perform the described processes are available at https://github.com/FatemehFN/consistent_groups_of_sms. To the best of our knowledge, it is the only python code that calculates of functional diversity for directed bipartite networks and suggests restoration strategies accordingly (Fig. 14).

## Data Availability

The identification of minimal trap spaces and stable motifs was performed using the python package pyboolnet available at https://github.com/hklarner/pyboolnet. The Logical Domain of Influence of the simplified Boolean models was calculated using the python package BooleanDOI available at https://github.com/yanggangthu/BooleanDOI. The identification of conditionally stable motifs, consistent groups of motifs and motif groups, and attractors was done using the python package available at https://github.com/FatemehFN/consistent_groups_of_sms.

## Code availability

The code developed in this study is also available at https://github.com/FatemehFN/consistent_groups_of_sms. It includes: (1) Control operations that contain the functions that identify the driver sets of stable motifs and conditionally stable motifs, and the functions that perform extinction and restoration of species and report the damage or restoration percentages. (2) Structure operations that contain functions that find the structural measures for each network. These include node nestedness, node MusRank score, Functional diversity and the corresponding restoration strategy. (3) Relationship operations that contain the functions that calculate the Logical Domain of Influence in threshold functions. (4) Example networks and the corresponding codes that illustrate the process by which one can reproduce the results in this study. The network ensembles are available from the corresponding authors upon reasonable request.
